# The Relationship between Inflammatory Biomarkers and Telomere Length in an Occupational Prospective Cohort Study

**DOI:** 10.1371/journal.pone.0087348

**Published:** 2014-01-27

**Authors:** Jason Y. Y. Wong, Immaculata De Vivo, Xihong Lin, Shona C. Fang, David C. Christiani

**Affiliations:** 1 Harvard School of Public Health, Department of Epidemiology, Boston, Massachusetts, United States of America; 2 Harvard School of Public Health, Department of Environmental Health, Boston, Massachusetts, United States of America; 3 Harvard School of Public Health, Department of Biostatistics, Boston, Massachusetts, United States of America; 4 Brigham and Women's Hospital, Channing Division of Network Medicine, Boston, Massachusetts, United States of America; 5 Massachusetts General Hospital, Boston, Massachusetts, United States of America; 6 Harvard Medical School, Department of Medicine, Boston, Massachusetts, United States of America; 7 New England Research Institutes, Watertown, Massachusetts, United States of America; I2MC INSERM UMR U1048, France

## Abstract

**Background:**

Chronic inflammation from recurring trauma is an underlying pathophysiological basis of numerous diseases. Furthermore, it may result in cell death, scarring, fibrosis, and loss of tissue function. In states of inflammation, subsequent increases in oxidative stress and cellular division may lead to the accelerated erosion of telomeres, crucial genomic structures which protect chromosomes from decay. However, the association between plasma inflammatory marker concentrations and telomere length has been inconsistent in previous studies.

**Objective:**

The purpose of this study was to determine the longitudinal association between telomere length and plasma inflammatory biomarker concentrations including: CRP, SAA, sICAM-1, sVCAM-1, VEGF, TNF-α, IL-1β, IL-2, IL-6, IL-8, and IL-10.

**Methods:**

The longitudinal study population consisted of 87 subjects. The follow-up period was approximately 2 years. Plasma inflammatory biomarker concentrations were assessed using highly sensitive electrochemiluminescent assays. Leukocyte relative telomere length was assessed using Real-Time qPCR. Linear mixed effects regression models were used to analyze the association between repeated-measurements of relative telomere length as the outcome and each inflammatory biomarker concentration as continuous exposures separately. The analyses controlled for major potential confounders and white blood cell differentials.

**Results:**

At any follow-up time, each incremental ng/mL increase in plasma CRP concentration was associated with a decrease in telomere length of −2.6×10^−2^ (95%CI: −4.3×10^−2^, −8.2×10^−3^, p = 0.004) units. Similarly, the estimate for the negative linear association between SAA and telomere length was −2.6×10^−2^ (95%CI:−4.5×10^−2^, −6.1×10^−3^, p = 0.011). No statistically significant associations were observed between telomere length and plasma concentrations of pro-inflammatory interleukins, TNF-α, and VEGF.

**Conclusions:**

Findings from this study suggest that increased systemic inflammation, consistent with vascular injury, is associated with decreased leukocyte telomere length.

## Introduction

Telomeres are nucleoprotein structures which act as vanguards against chromosomal decay. The repetitive (TTAGGG)_n_ sequences of telomeres are located at the distal ends of eukaryotic chromosomes and span 8–12 kb at birth [1]. Erosion of telomeres below critical lengths may lead to aberrant recombination, chromosomal fusion, and even subsequent neoplasia. Indeed, numerous epidemiological investigations have implicated diminished telomere length with increased risk of adverse health outcomes. For instance, previous studies have reported that shorter telomeres were significantly associated with increased overall cancer risk, in addition to decreased survival rate amongst diagnosed cases [2]–[4]. Furthermore, truncated telomeres have been found to be associated with increased risk of cardiovascular diseases [5]. Telomeres truncate by 30–200 bp with every mitotic division as a consequence of intrinsic limitations in lagging-strand DNA synthesis [6]. As a result, telomere length may provide a portrait of the mitotic history of a cell. Maintaining sufficient telomere length involves an interplay between factors which erode telomeres, and factors which extend them. Extension of telomeres can be accomplished via activity of the ribonucleoprotein telomerase. Although constitutively expressed in germ line tissue and omnipotent stem cells, telomerase is only basally expressed in most somatic tissue; thus resulting in gradual decay of telomeric repeats. Aside from cellular division, telomeric shortening can be further intensified by extrinsic environmental factors which induce oxidative stress under conditions of inflammation [6], [7]. Therefore, telomere length may also be viewed as a marker that is reflective of cellular coping; providing resilience against biochemically-induced genomic trauma.

Inflammation is a complex physiological response to deleterious stimuli such as injury and infection. The inflammatory process may be acute as a result of transient stimuli, or may persist as a chronic state as a result of continual exposure. Chronic inflammation is an underlying pathophysiological basis of numerous diseases such as cancer, myocardial infarction, and lung failure [8]. With recurrent injury to tissue, subsequent cell death, scarring, and fibrosis may lead to permanent loss of function [8].

Chronic inflammation may result in both cellular apoptosis and necrosis; facilitating the release of potent reactive oxygen species (ROS) such as superoxides, hydroxyl radicals, and hydrogen peroxide from mitochondria into the vicinity [9]–[11]. These oxidizing agents can induce genomic trauma such as double-stranded breaks and chemical changes to the telomeric sequences of DNA. Indeed, previous studies found that exposure to airborne particulate matter is associated with increased oxidative damage to the genome, as reflected by urinary concentrations of 8-hydroxydeoxyguanosine (8-OHdG), an oxidized nucleoside of DNA [12]. Furthermore, oxidative stress has also been shown to produce chemical alterations to purines and pyrimidines of telomeric oligonucleotides in the form of 8-oxoguanine adducts [13]. Should these chemical alterations occur *in vivo*, inhibition of the interaction between Telomeric repeat-binding factors (TRF1/TRF2) and telomeric sequences may produce instability of the shelterin loop complex; potentially leading to telomeric erosion, dysregulation of gene expression, chromosomal fusion, and disruption of cellular function [14].

Recent studies found that prolonged exposure to particulate air pollution increases levels of acute phase proteins and vascular injury related inflammatory mediators including C-Reactive Protein (CRP), serum intercellular adhesion molecule-1 (sICAM-1), and serum vascular cell adhesion molecule-1 (sVCAM-1); important biomarkers of cancer and cardiovascular disease [15], [16]. Additionally, inflammation may trigger the release of interleukins (IL); a subset of cytokines which can produce highly variable physiological and immune responses in different cell types [17]. In particular, Interleukin-1 (IL-1) is a pro-inflammatory cytokine primarily produced by macrophages, that mediates inflammatory diseases by initiating and potentiating inflammatory responses and hematopoiesis [18]. Interleukin-8 (IL-8) is an equally important cytokine produced by macrophages, which acts as a chemo-attractant for neutrophils during acute inflammatory responses. Moreover, IL-8 has been shown induce rapid mobilization of hematopoietic stem cells (HSC) and subsequent differentiation into leukocytes. This attribute of IL-8 may have important implications with respect to telomere erosion since increased mitotic division is required to replenish mobilized HSC [19]. Although inflammation and immune response may be responsible for both cytokine production and telomeric erosion, a number of interleukins including IL-2, IL-4, IL-6, and IL-10 have been shown to increase telomerase activity *in vitro* which may maintain telomere length; illustrating the complexity of these molecular interactions [14], [15].

Despite numerous studies showing the association between telomere length and various chronic diseases, the intricate causal relationship between these variables has been difficult to unravel. The association between telomere length and disease may be driven by an unmeasured common cause (confounder), such as underlying chronic inflammation. Although the association between inflammatory markers and chronic health outcomes such as cardiovascular disease has been firmly established, the relationship between these markers and telomere dynamics is less defined. IL-6, CRP, and Tumor Necrosis Factor (TNF-α) have been found in several studies to be associated with decreased telomere length [20] [21] [22]. Although well-powered, most of these studies have been of cross-sectional design. Investigation into the biological interplay between these molecular markers over time in a longitudinal setting would further add to the understanding of the etiological basis of chronic diseases. Therefore, the objective of this study was to determine the longitudinal associations between telomere length and plasma inflammatory biomarker concentrations including: CRP, Serum Amyloid A (SAA), sICAM-1, sVCAM-1, Vascular Endothelial Growth Factor (VEGF), TNF-α, IL-1β, IL-2, IL-6, IL-8, and IL-10. Furthermore, we explored whether these inflammatory biomarkers modify the rate of telomeric change over a 2 year follow-up period. We hypothesize that increased levels of pro-inflammatory biomarkers is correlated with decreased telomere length, and intensifies the rate of erosion over the follow-up period. The prospective determination of plasma inflammatory mediators and telomere length at multiple time points is particularly important in addressing numerous gaps in current knowledge, including the dynamic relationship between inflammation, immune response, and genomic trauma.

## Materials and Methods

### Study Design

**Figure 1:**
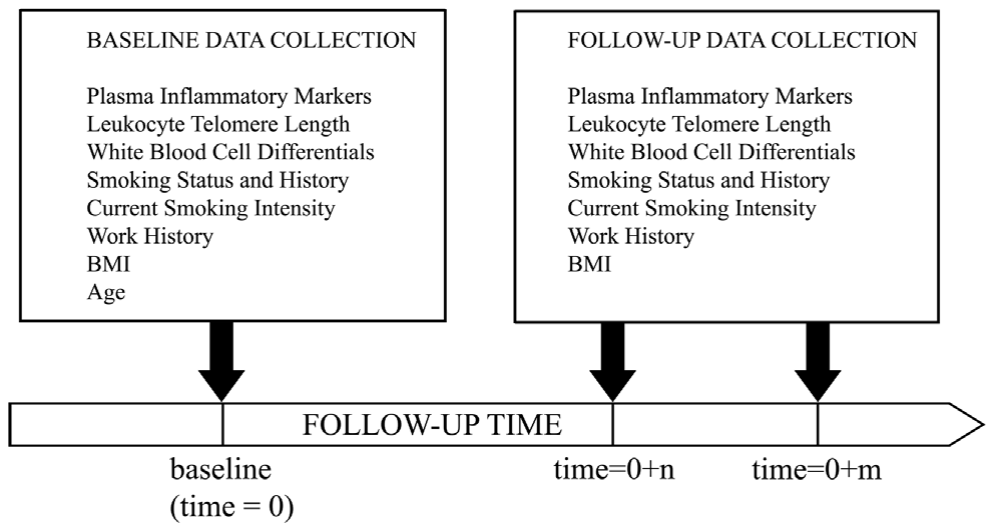
Repeated-Measures Data Collection of the Harvard Boilermakers Longitudinal Study.

The Harvard Boilermakers Longitudinal Study is a prospective open-cohort comprised of a series of periodic short-term panel studies. The source population consists of members of the International Brotherhood of Boilermakers Union, Local 29 in Quincy, MA. There are currently 400 active union members, 190 of which have been prospectively followed since 1999. Their primary occupational activity is welding, assembling, and repairing boilers that provide high-pressure steam to drive electrical turbines in power plants. Therefore, they are exposed to environments with high-levels of occupational fine particulate matter (PM_2.5_). The follow-up period for the current longitudinal study was from January 29^th^ 2010 to June 16^th^ 2012. The current study population consisted of a panel of 87 subjects at baseline. The inclusion criteria for this study were being male union members, apprentices or journeymen, and over 18 years of age at the time of recruitment. The timescale of interest was days of follow-up, while repeated measures of the outcome and predictors were obtained at irregularly spaced time intervals between baseline blood draw and the end of follow-up (Figure 1). A total of 350 inflammatory biomarker measurements and 244 telomere measurements were obtained for the 87 subjects, with an average of 3 measurements per subject. One subject diagnosed with arthritis and having a history of myocardial infarction was excluded from the analysis.

### Ethics Statement

This observational study was approved the by Institutional Review Board at the Harvard School of Public Health (OHRA Protocol #: P10444, Cardiopulmonary Response to Particulate Exposure, PI: David C. Christiani). Informed written consent was obtained from all subjects prior to inclusion in the study.

### Exposure Assessment

#### Vascular Injury Biomarkers and Pro-inflammatory Cytokines

Whole blood samples were drawn from subjects by a certified phlebotomist into collection tubes containing EDTA. Plasma was isolated from whole blood by centrifugation at 1200 RPM for 12 minutes. Plasma samples were then aliquoted into cryogenic tubes and stored at −80°C. The number of freeze-thaw cycles prior to the assay was limited to one. Plasma concentrations of CRP, SAA, sICAM-1, and sVCAM-1 were assessed via multiplex electrochemiluminescence using the MULTI-SPOT® 96-well Human Vascular Injury Panel II assay (Meso Scale Discovery, Rockville MD) as per manufacturer's protocol. Similarly, plasma concentrations of IL-1β, IL-2, IL-6, IL-8, IL-10, TNF-α, and VEGF were assessed using the 7-plex MULTI-ARRAY® 96-well custom cytokine/chemokine assay. Duplicate reactions were performed on the same 96-well plate, each using 10 µL of plasma. Repeated measurements over time for each individual subject were performed on the same 96-well plate. MULTI-SPOT® vascular injury plates contained an 8-point standard curve from 0–1000 ng/mL with 7-fold dilutions, in order to derive inflammatory marker concentration. The MULTI-ARRAY® cytokine plates contained an 8-point standard curve from 0–2500 pg/mL with 4-fold dilutions. Electrochemiluminescent signals from the MULTI-SPOT® and MULTI-ARRAY® assay plates were read using a SI2400A Sector Imager (Meso Scale Discovery, Rockville MD). Three quality control samples were interspersed through-out all assay plates in duplicate. The average within-plate % coefficients of variation (%CVs) ranged from 7.6%–10.9% for the different assays. The average between-plate %CVs ranged from 12.3%–17.4% for the different assays.

### Outcome Assessment

#### Telomere Real-Time Quantitative PCR

Buffy coat was extracted from whole blood and stored in cell lyses solution at −20°C until DNA extraction. Peripheral blood leukocyte DNA was extracted using the QIAmp DNA blood kits (QIAGEN, Venlo, Netherlands). Average relative telomere length was assessed using Real-Time Quantitative PCR (qPCR) [23]. This assay determines the copy-number ratio between telomeric repeats and a single-copy (36B4) reference gene (T/S Ratio, -dCt). The T/S ratio is reflective of the average telomere length across all chromosomes in a population of cells and was calculated for each participant by subtracting the average 36B4 threshold cycle (Ct) value from the average telomere Ct value. The T/S ratio value for all experimental samples was then compared to the T/S ratio of a reference sample, consisting of a pooled genomic DNA sample. The relative T/S ratio (-ddCt) was determined by subtracting the T/S ratio of the reference sample from the T/S ratio of each unknown sample, and then exponentiating (2^−ddCt^). A modified version of the qPCR telomere assay was performed in a 384-well format with a 7900HT PCR System (Life Technologies, Carlsbad, CA). Briefly, 5 ng of buffy-coat derived genomic DNA was dried down in a 384-well plate and resuspended in 10 µL of either the telomere or 36B4 reaction mixture for 2 hours at 4°C. The telomere reaction mixture consisted of 1× Quantitect SYBR Green Master Mix (Qiagen, Venlo, Netherlands), 2.5 mM of DTT, 270 nM of Tel-1 primer-(GGTTTTTGAGGGTGAGGGTGAGGGTGAGGGTG AGGGT), and 900 nM of Tel-2 primer-(TCCCGACTATCCCTATCCCTATCCCTATCCCTATCCCTA). The reaction proceeded for 1 cycle at 95°C for 5 min, followed by 40 cycles at 95°C for 15 sec, and 54°C for 2 min. The 36B4 reaction consisted of 1× Quantitect SYBR Green Master Mix, 300 nM of 36B4U primer-(CAGCAAGTGGGAAGGTGTAATCC), and 500 nM of 36B4D primer- (CCCATTCTATCATCAACGGGTACAA). The 36B4 reaction proceeded for 1 cycle at 95°C for 5 min, followed by 40 cycles at 95°C for 15 sec, and 58°C for 1 min 10 sec. All samples for both the telomere and 36B4 reactions were performed in triplicate on different plates. Each 384-well plate contained a 6-point standard curve from 0.625 ng to 20 ng to assess PCR efficiency. The slope of the standard curve for both the telomere and 36B4 reactions was −3.40+/−0.15. Quality control samples were interspersed throughout the plates in order to assess inter-plate and intra-plate variability of Ct values. The inter-plate %CVs for both the telomere Ct and 36B4 Ct values were under 0.75%. Multiple relative telomere length measurements for each subject within the same unit of follow-up time (day) were dropped at random.

### White Blood Cell Differentials

Complete blood counts including leukocyte differentials were assessed using a XE-2100™ Automated Hematology System (Sysmex, Kobe, Japan) by Laboratory Corporation of America Holdings (LabCorp) (Newton, MA). Previous studies have suggested telomere lengths vary between different leukocyte subtypes [24], [25]. Therefore, telomere length assessment may be affected if the compositional proportion of these subtypes is altered. This augmentation in proportion may not represent a true change in the telomere length of a given cell, but rather reflects a change in the population of cells within a given sample. The change in proportion could be due to infection or injury during blood draw, immunologic abnormalities, or inflammatory conditions. Therefore, it was prudent to control for white blood cell differentials in the analysis.

### Covariate Assessment

Self-reported questionnaires were used to collect demographic data such as birthdate, sex, race, smoking status, current smoking intensity, and work history. Number of years as a boilermaker was used as a surrogate variable for long-term particulate matter exposure from welding fumes [26], [27]. Anthropometric data such as height and weight used to derive body mass index (BMI) were measured by research personnel at the study site.

### Analysis

#### Linear Mixed Models for Longitudinal Analysis

Linear mixed effects regression models with random intercept were used to estimate the association between repeated measures of relative telomere length as the outcome, and plasma inflammatory biomarker concentrations as continuous exposures. Separate models were used for each inflammatory marker. The models also controlled for covariates including log-transformed number of years as a boilermaker at baseline (continuous), time-varying log-transformed BMI (continuous, log kg/m^2^), age at baseline blood draw (continuous), current smoking intensity (cigarettes per day), and white blood cell differentials including neutrophil %, lymphocyte %, monocyte %, and eosinophil %. Basophil proportions were not controlled for because of miniscule percentages and lack of variability. Interaction terms between the exposure and follow-up time were included to explore effect modification of the rate of telomeric change. Analysis was implemented in SAS v9.3 using PROC MIXED. In order to obtain unbiased estimates with a limited sample size, restricted maximum likelihood (REML) was used to estimate the coefficients and variance-covariance matrices.

#### Adjustment for Family-Wise Error Rate

Separate linear mixed effects models were used to estimate the association between each inflammatory biomarker and telomere length. Each inflammatory biomarker was classified into one of two families because of differences of effects in the inflammatory process, vast disparities in plasma concentration ranges, and assessment on different assay platforms. The first family consisting of CRP, SAA, sICAM-1, and sVCAM-1, were vascular injury markers measured in the ng/mL range using the quadraplex MULTI-SPOT® assay. The second family consisting of IL-1β, IL-2, IL-6, IL-8, IL-10, VEGF, and TNF-α, were inflammatory cytokines/chemokines assessed in the pg/mL range using the 7-plex MULTI-ARRAY® assay. Therefore, Bonferroni adjusted α-values of 0.013 and 0.007 were used to control family-wise error rate in the vascular injury family and inflammatory cytokine family respectively. Although Bonferroni correction was first developed under the assumption of independence between tests, it can also be used in situations where the tests maybe dependent as with plasma biomarkers. When multiple tests are dependent, Bonferroni correction is considered to be “conservative” in that the true family-wise risk of a type-I error (false-positive) is less than the stated family-wise α [28], [29]. In other words, when the assumption of independence is violated, Bonferroni adjustment still protects well against false-positives, but has less power to detect true effects [28], [29].

#### Management of Missing Data

The study population was an open cohort with staggered entry and loss of follow-up. Furthermore, data collection occurred at irregularly spaced time intervals. Therefore, outcome and predictor data was unbalanced across the time points. Linear mixed models do not require outcome data to be balanced nor collected at regularly spaced intervals because they use maximum likelihood (ML) or REML to estimate the coefficients and covariance structures; allowing all available data to be used [30]. The estimates are valid under the assumption that data are missing at random (MAR) and distributional assumptions are correct [30]. However, verification of MAR assumptions is nearly impossible in epidemiological studies [31]. Inflammatory biomarker readings below the lowest point of the linear range of the standard curve were assigned half the value of the minimum detection limit and retained for analysis [32], [33].

## Results

### Baseline Study Population Characteristics

The study population was composed of 87 male subjects. The subjects were 86.1% self-identified white, whereas the remaining subjects were self-identified as African-American, Hispanic, and Asian (Table 1). One-way ANOVA analysis indicated no statistical difference in telomere length between self-reported racial groups. Self-identified race was not controlled for in the analysis because there was no statistical difference between categories and the majority of subjects were of white heritage. Current smokers composed 35.6% and ever-smokers composed 54.0% of the study population at baseline. A two-sample Student's t-test indicated no statistical difference in telomere length between smokers and non-smokers. Furthermore, there was no statistical difference in telomere length between ever-smokers and never smokers. However, current smoking intensity was included as a confounder in subsequent analysis because of its established causal relationship with telomere length [34]–[36]. The average age at baseline was 41.5+/−13.0SD years; having worked an average of 11.1+/−10.4SD years as a boilermaker. The average BMI was 28.4+/−4.8 kg/m^2^.

**Table 1:** Characteristics of the Harvard Boilermakers Longitudinal Study Population at Baseline.

Variable	Total n = 87		Mean Relative		
	Frequency	(%)	Telomere Length	Std. Dev.	p-value ‡
Current Smoking Status					
Non-smoker	52	59.8	0.60	0.18	0.22
Smoker	31	35.6	0.66	0.17	
Past Smoking Status					
Never	17	19.5	0.63	0.22	0.99
Ever	47	54.0	0.63	0.17	
Self-Reported Race					
White	71	81.6	0.62	0.17	0.71
African-American	7	8.0	0.61	0.22	
Hispanic	3	3.4	0.66	0.28	
Asian	2	2.3	0.76	0.11	
	**Mean**	**Std. Dev.**	**Median**	**IQR**	
Age	41.5	13.0	40.0	31.4–51.5	
Number of Years Working as a Boilermaker	11.1	10.5	9.0	3.0–11.5	
Body Mass Index (BMI) (kg/m^2^)	28.4	4.8	27.3	25.2–32.4	
Relative Telomere Length (2^−ddCt^)	0.63	0.18	0.60	0.5–0.7	
Plasma C-Reactive Protein (CRP) Conc. (ng/mL)	4790	7602	2740	896–6064	
Plasma Serum Amyloid A (SAA) Conc. (ng/mL)	5636	2881	8887	1260–6722	
Plasma sICAM-1 Conc. (ng/mL)	358	121	346	282–426	
Plasma sVCAM-1 Conc. (ng/mL)	531	181	527	406–628	
Plasma VEGF Conc. (pg/mL)	380	237	343	148–469	
Plasma TNF-α (pg/mL)	12.2	12.4	11.0	8.2–13.2	
Plasma Interleukin-1 (IL-1β) Conc. (pg/mL)	1.3	2.5	0.5	0–1.4	
Plasma Interleukin-2 (IL-2) Conc. (pg/mL)	4.4	14.4	2.0	1.0–3.4	
Plasma Interleukin-6 (IL-6) Conc. (pg/mL)	25.2	20.9	18.3	12.2–28.6	
Plasma Interleukin-8 (IL-8) Conc. (pg/mL)	17.7	17.0	14.6	10.0–19.8	
Plasma Interleukin-10 (IL-10) Conc. (pg/mL)	7.0	7.0	5.5	4.0–7.6	

‡ A two-sample Student's T-Test was used to compare telomere length in covariates with two categories (t-statistic), whereas a one-way ANOVA was used to evaluate covariates with three or more categories (F-statistic). *p-values≤0.05 were considered statistically significant.

Discrepancies in counts due to missing data.

### Linear Mixed Models: Longitudinal Analysis of the Association between Vascular Injury Related Inflammatory Markers and Telomere Length

After Bonferroni adjustment of α-levels, statistically significant negative associations were found between telomere length and plasma concentrations of both CRP and SAA (Table 2). At any follow-up time, each incremental ng/mL increase in CRP concentration was associated with a decrease in telomere length of −2.6×10^−2^ (95%CI: −4.3×10^−2^, −8.2×10^−3^, p = 0.004) units, while controlling for other covariates. Similarly, the estimate for the negative linear association between SAA and telomere length was −2.6×10^−2^ (95%CI:−4.5×10^−2^, −6.1×10^−3^, p = 0.011). None of the interaction terms between follow-up time and vascular injury inflammatory markers were significant. Although the main effects for sICAM-1 and sVCAM-1 on telomere length were not statistically significant after adjustment for family-wise error rate, they were marginally significant with an unadjusted α-level of 0.05. As with CRP and SAA, the effect estimates of −2.4×10^−2^ (95%CI: −4.8×10^−2^, −9.0×10^−5^, p = 0.049) for sICAM-1 and −2.4×10^−2^(95%CI: −4.6×10^−2^, −2.1×10^−3^, p = 0.032) for sVCAM-1 indicated negative linear associations with telomere length. The rate of telomeric decay had an approximate range of 0.0007–0.0015 units per day, at any concentration of the vascular injury markers and controlling for predictors.

**Table 2:** The Association between Vascular Injury Inflammatory Markers and Telomere Length.

C-Reactive Protein (CRP)	Estimate	95%CI Lower	95%CI Upper	p-value	
Main Effect (log ng/mL)	−2.6 x10^−2^	−4.3 x10^−2^	−8.2 x10^−3^	0.004	*
Follow-up time (days)	−6.9 x10^−4^	−1.4 x10^−3^	2.6 x10^−5^	0.059	
Main Effect x Follow-up time	4.8 x10^−5^	−4.0 x10^−5^	1.4 x10^−4^	0.301	
**Serum Amyloid A (SAA)**					
Main Effect (log ng/mL)	−2.6 x10^−2^	−4.5 x10^−2^	−6.1 x10^−3^	0.011	*
Follow-up time (days)	−8.8 x10^−4^	−1.7 x10^−3^	−6.0 x10^−5^	0.036	
Main Effect x Follow-up time	6.9 x10^−5^	−3.0 x10^−5^	1.7 x10^−4^	0.176	
**Intercellular Adhesion Molecule (sICAM-1)**					
Main Effect (log ng/mL)	−2.4 x10^−2^	−4.8 x10^−2^	−9.0 x10^−5^	0.049	
Follow-up time (days)	−1.5 x10^−3^	−2.5 x10^−3^	−5.6 x10^−4^	0.002	*
Main Effect x Follow-up time	2.1 x10^−4^	4.1 x10^−5^	3.8 x10^−4^	0.015	
**Vascular Cell Adhesion Molecule (sVCAM-1)**					
Main Effect (log ng/mL)	−2.4 x10^−2^	−4.6 x10^−2^	−2.1 x10^−3^	0.032	
Follow-up time (days)	−1.5 x10^−3^	−2.4 x10^−3^	−5.2 x10^−4^	0.003	*
Main Effect x Follow-up time	1.8 x10^−4^	3.1 x10^−5^	3.3 x10^−4^	0.019	

Separate linear mixed models were used for each inflammatory marker. Models controlled for white blood cell count, neutrophil %, lymphocyte %, monocyte %, eosinophil %, current smoking intensity (cigarettes per day), age at baseline blood draw (years), BMI (log kg/m^2^), and years as a boilermaker (log years). Main effects were log-transformed to achieve a normal distribution.*p-values below the Bonferroni-adjusted α-level of 0.013 were considered statistically significant.

The estimate for the main effect represents the difference in telomere length per incremental ng/mL increase in the inflammatory marker at any follow-up time, controlling for other predictors. The estimate for follow-up time represents the rate of telomeric change; the difference in telomere length per day of follow-up when all other predictors are at zero. The estimate for the main effect x follow-up time interaction represents effect modification of the rate of telomeric change by the inflammatory marker; the change in telomere length per ng/mL increase in the inflammatory marker over each day of follow-up time, holding other predictors constant. 95% Confidence Intervals (95%CI) are the bounds in which with more independent samplings, the true estimate will fall within this range in 95% of those samplings.

### Linear Mixed Models: Longitudinal Analysis of the Association between Inflammatory Cytokines/Chemokines and Telomere Length

After adjustment for family-wise error rate, no statistically significant associations were found between telomere length and the panel of inflammatory cytokines/chemokines composed of IL-1β, IL-2, IL-6, IL-8, IL-10, TNF-α, and VEGF (Table 3). Furthermore, none of the interaction terms between follow-up time and inflammatory cytokines/chemokines were significant. The rate of telomeric attrition had an approximate range of 0–0.0006 units per day, at any concentration of the inflammatory cytokines and controlling for predictors.

**Table 3:** The Association between Inflammatory Cytokines-Chemokines and Telomere Length.

Interleukin-1β (IL-1β)	Estimate	95%CI Lower	95%CI Upper	p-value	
Main Effect (log pg/mL)	1.7 x10^−2^	−2.4 x10^−2^	5.8 x10^−2^	0.409	
Follow-up time (days)	−3.3 x10^−4^	−4.8 x10^−4^	−1.8 x10^−4^	<0.0001	*
Main Effect x Follow-up time	1.1 x10^−5^	−1.7 x10^−4^	1.9 x10^−4^	0.903	
Interleukin-2 (IL-2)					
Main Effect (log pg/mL)	−1.3 x10^−3^	−3.9 x10^−2^	3.7 x10^−2^	0.948	
Follow-up time (days)	−3.1 x10^−4^	−5.1 x10^−4^	−1.0 x10^−4^	0.003	*
Main Effect x Follow-up time	−4.0 x10^−5^	−2.3 x10^−4^	1.5 x10^−4^	0.661	
Interleukin-6 (IL-6)					
Main Effect (log pg/mL)	3.8 x10^−2^	−8.8 x10^−3^	8.4 x10^−2^	0.110	
Follow-up time (days)	1.5 x10^−5^	−5.9 x10^−4^	6.2 x10^−4^	0.959	
Main Effect x Follow-up time	−1.2 x10^−4^	−3.1 x10^−4^	7.2 x10^−5^	0.219	
Interleukin-8 (IL-8)					
Main Effect (log pg/mL)	2.0 x10^−2^	−2.6 x10^−2^	6.5 x10^−2^	0.397	
Follow-up time (days)	1.0 x10^−5^	−4.8 x10^−4^	5.0 x10^−4^	0.968	
Main Effect x Follow-up time	−1.3 x10^−4^	−3.1 x10^−4^	4.6 x10^−5^	0.146	
Interleukin-10 (IL-10)					
Main Effect (log pg/mL)	−2.5 x10^−2^	−6.9 x10^−2^	2.0 x10^−2^	0.269	
Follow-up time (days)	−6.0 x10^−4^	−9.7 x10^−4^	−2.2 x10^−4^	0.002	*
Main Effect x Follow-up time	1.3 x10^−4^	−4.0 x10^−5^	2.9 x10^−4^	0.133	
Vascular Endo. Growth Factor (VEGF)					
Main Effect (log pg/mL)	1.4 x10^−2^	−1.8 x10^−2^	4.7 x10^−2^	0.383	
Follow-up time (days)	−2.5 x10^−4^	−1.1 x10^−3^	6.4 x10^−4^	0.581	
Main Effect x Follow-up time	−2.0 x10^−5^	−1.7 x10^−4^	1.4 x10^−4^	0.836	
Tumor Necrosis Factor (TNF-α)					
Main Effect (log pg/mL)	2.7 x10^−3^	−4.5 x10^−2^	5.1 x10^−2^	0.911	
Follow-up time (days)	−3.1 x10^−4^	−8.6 x10^−4^	2.4 x10^−4^	0.261	
Main Effect x Follow-up time	−1.0 x10^−5^	−2.3 x10^−4^	2.1 x10^−4^	0.927	

Separate linear mixed models were used for each cytokine/chemokine. Models controlled for white blood cell count, neutrophil %, lymphocyte %, monocyte %, eosinophil %, current smoking intensity (cigarettes per day), age at baseline blood draw (years), BMI (log kg/m^2^), and years as a boilermaker (log years). Main effects were log-transformed to achieve a normal distribution. *p-values below the Bonferroni-corrected α-level of 0.007 were considered statistically significant.

The estimate for the main effect represents the difference in telomere length per incremental pg/mL increase in the cytokine/chemokine at any follow-up time, controlling for other predictors. The estimate for follow-up time represents the rate of telomeric change; the difference in telomere length per day of follow-up when all other predictors are at zero. The estimate for the main effect x follow-up time interaction represents effect modification of the rate of telomeric change by the cytokine/chemokine; the change in telomere length per pg/mL increase in the cytokine/chemokine over each day of follow-up time, holding other predictors constant. 95% Confidence Intervals (95%CI) are the bounds in which with more independent samplings, the true estimate will fall within this range in 95% of those samplings.

## Discussion

This is one of the few longitudinal studies which examined the relationship between expression levels of an extensive panel of pro-inflammatory markers and telomere length dynamics. Furthermore, the prospective study design incorporates a recent methodological shift in approaching the study of telomeres; evaluating both telomere length and the rate of change, as opposed to treating telomeres as static entities in epidemiological studies. The primary finding of this study was that increased plasma CRP and SAA concentrations were associated with decrease leukocyte telomere length. Thus far, no other epidemiological study has examined the association between SAA and telomere length. However, the results with respect to CRP were in concordance with findings from previous cross-sectional studies. The current study focused on an occupational cohort of mostly healthy, middle-aged, white-American men. In contrast, the previous Ten Town Heart Health Study of European and South Asian adolescents ages 13–16 years found that for every unit increase in CRP concentration, there was a 0.026 (95%CI: 0.011, 0.041, p<0.001) unit decrease in telomere length [22]. In the Nova Scotia Health Survey of 1995, those in the lowest tertile of telomere length (4.1–5.0 kb) had significantly higher levels of serum CRP levels compared to those in the middle (5.0–5.5 kb) and highest tertile of telomere length (5.5–8.2 kb) p-trend = 0.005 [37]. Furthermore, the Cardiovascular Health Study found a similar negative association between CRP concentration and telomere length with an estimate of −0.059+/−0.003SE, p = 0.02 [20].

CRP is a non-specific acute phase protein produced in the liver [38]. CRP is typically undetected in the plasma of healthy individuals; however, its concentration dramatically rises with localized and systemic inflammation. Although expressed during inflammatory response, CRP is not informative with respect to specific locations of injury [38]. The physiological purpose of CRP is to bind to phosphocholine expressed on the outer plasma membrane of apoptotic and necrotic cells to activate the complement system and subsequent phagocytosis [39]. Furthermore, cells undergoing apoptosis and necrosis experience genomic degradation mediated by caspase-activated and waste-management DNA nucleases, respectively [40]. Apoptotic and necrotic cells can also release reactive oxygen species from their mitochondria which can further induce oxidative damage to the telomeres of neighboring cells. Therefore, the negative correlation between increased plasma CRP and decreased telomere length may be attributed to the common cause of inflammation, mediated through oxidative cell damage. However, the cytotoxicity of the prominent occupational exposure (particulate matter from welding fumes) on the target tissue was not assessed due to the obvious practical limitations of population-based epidemiological studies. However, PM_2.5_ has been shown in observational studies to be associated with increased inflammatory response, in addition to being shown in laboratory studies to induce cytotoxic cell death [41] [42] [43].

Serum Amyloid A is also an acute phase protein which is elevated during acute and systemic inflammation. SAA is primarily produced by the liver and is associated with high-density lipoprotein (HDL) in the circulation [44]. However, the exact *in vivo* functions of SAA are still not well defined [45].

The association between SAA and telomere length may be driven the common cause of adiposity.

SAA is also produced by adipocytes and is correlated with obesity and insulin resistance [46]. Adipose cells also produce endogenous 17β-estradiols which can act as potent mitogens. Therefore, increased estradiol-induced mitotic division of HSC may result in increased telomere erosion in leukocytes [47]. Furthermore, increased adiposity has also been linked with elevated vascular injury and inflammation, for which SAA is a marker [48]. Inflammation from adiposity-related vascular injury may cause apoptotic or necrotic cell death, and subsequent telomere shortening by the aforementioned mechanisms. BMI is a convenient measure which is commonly used in epidemiological studies to control for confounding by adiposity. Although BMI was controlled in the analysis, it is not a perfect surrogate for degree of adiposity [49]. The median BMI of the study population was high, but from a qualitative standpoint, it was not fully representative of adiposity due to the muscular nature of the boilermaker subjects. Therefore, there may be residual confounding by adiposity in the relationship between inflammation and telomere length. However, the estimate was quite significant even after Bonferroni-adjustment, which would suggest that the degree of residual confounding would have to be substantial in order to explain the observed association. Given the direction and magnitude of the observed association between telomere length, CRP, sICAM-1, and sVCAM-1; it was more likely that vascular-injury related inflammation, for which SAA is another marker, explains more of the variability in telomere length than residual confounding by adiposity.

In contrast to the vascular injury marker panel, no significant associations between inflammatory IL, chemokines, and telomere length were found in this study. Previous studies found significant relationships between these molecular markers. In particular, the Health, Aging, and Body Composition Study found that cumulative inflammatory load, as indexed by the combination of high levels of IL-6 and TNF-α, was associated with increased odds for short leukocyte telomeres [21]. Furthermore, a study of renal cancer found significant positive correlations between tumor TL and peripheral levels of IL-8 and IL-10 [50]. There may be both biological and methodological explanations for the discrepancy in findings between the studies. As previously mentioned, inflammation may be responsible for both IL production and telomeric erosion; however, interleukins including IL-2, IL-4, IL-6, and IL-10 have been shown to increase telomerase activity, thus maintaining telomere length [51], [52]. Since the activity of various IL is diverse, the biological effects of each IL may counteract each other. Furthermore, differences in the underlying exposure profiles and inflammatory conditions between the subjects of various studies may induce production of different IL and/or differing amounts of IL. Indeed, the distribution of exposures and demographic characteristics such as PM_2.5_ exposure, age, gender, socioeconomic status, and race were substantially different across studies. Additionally, there were significant differences in study design, exposure assessment and operationalization, outcome assessment, and analysis between studies. In regards to our study, the variability in plasma IL concentrations may have been too narrow to drive variability in telomere length. In contrast, the plasma concentrations of vascular injury markers were much broader. Although the statistical power of this study was substantially increased with the use of repeated-measures, the sample size may not have been sufficient to compensate for the narrow variability of the plasma IL.

Interestingly, none of the interaction terms between follow-up time and inflammatory markers examined in this study reached statistical significance. These null-findings are consistent with the hypothesis that immune response does not alter the rate of telomeric decay. However, caution should be taken when interpreting these findings, because the limited sample size and follow-up duration of this study precludes definitive evaluation of effect modification of rates. Although repeated-measures increase the power to detect subtle effects, there was a possibility that the null interactions may be non-informative. Furthermore, previous studies have shown that the rate of telomeric decay is greatest during early developmental stages of rapid growth between 0–10 years of age; whereas the rate of attrition significantly decreases into post-adolescence and adulthood [53]. Indeed, only miniscule rates of attrition were observed over a 2 year period in the current study of adults. Therefore, incredibly substantial effects would be required in a short follow-up period to drive a truly significant interaction term, which would be difficult even in studies with large sample sizes with long follow-up duration.

The most prominent strength of this study was its prospective design which allowed for improved assessment of the temporality between exposure, outcome, and covariates. Longitudinal analysis using mixed effects models allows for the incorporations of time-varying exposures, outcomes, and covariates; which permits exploration of the rates of change. Moreover, the longitudinal design enhances analytical control of confounding. Additionally, the study design imparts robustness against recall bias of the exposure and differential misclassification; as both the exposure and outcome were independent laboratory measurements. Moreover, the use of multiple repeated-measures throughout the follow-up period substantially increased the statistical power to detect subtle effects, beyond the apparent sample size of the study. Notably, exposure assessment was accomplished using highly sensitive electro-chemiluminescent assays on an extensive panel of eleven inflammatory markers. The Meso Scale Discovery platform offers superior dynamic detection ranges, accuracy, and reproducibility compared to traditional ELISA, which allows for more nuanced analyses of the relationship between inflammatory biomarkers and telomere length [54]. Furthermore, the outcome was assessed using qPCR which is currently the most labor- and cost-effective methods to measure telomere length in epidemiological studies. Not only does qPCR correlate well with gold-standard Southern blots, but also requires only a fraction of the genomic DNA, and is robust against detection of centromeric sequences [23].

As with all scientific investigations, there were limitations of note. The practical restrictions of occupational epidemiology studies involving real human subjects creates complexities in the study design and analysis, which requires nuanced appreciation to evaluate. For instance, union policies and seasonal work-training schedules of the boilermaker subjects created a situation in which entry and exit from the study was staggered, therefore data was unbalanced. Fortunately, analysis of longitudinal data using versatile linear mixed models does not require balanced data under the aforementioned assumptions. There may be the possibility of bias due to unmeasured confounders. However, most notable confounders related to inflammatory markers and telomere length from prior studies were controlled for in the analysis. Although controlling for time-varying confounders in traditional models may result in selection bias, the degree of bias would depend on the true state-of-affairs between all the related variables; which is inherently unknowable [55]. As with most occupational cohorts, the sample size of this study was limited. However, the entire source population from which the subjects were recruited was only 400 union members; therefore our current study population contains a significant proportion of the entirety. The use of multiple repeated-measures should compensate for this limitation by significantly increasing the statistical power to detect small effect sizes. There was also the possibility of healthy worker bias (selection bias) in which healthier subjects with lower inflammation, and longer telomeres tended to be recruited and/or remain in the study compared to others. Healthy worker recruitment bias would reduce generalizability of the results to the source population. Healthy worker selection bias, a form of loss of follow-up, would likely bias the estimates towards the null and underestimate the true effect. Lastly, the linear mixed models may have been over-adjusted because numerous covariates and white blood cell differentials needed to be controlled in order to minimize bias in the estimates. Although the variance may have been inflated from over-fitting, statistically significant estimates were still obtained for the analysis involving vascular injury markers and telomere length.

In summary, this study provides an enticing glimpse into the relationship between systemic inflammation, immune response, and genomic degeneration. Significant negative correlations were found between telomere length and plasma concentrations of CRP and SAA. Furthermore, marginal yet insignificant negative associations were found between telomere length, and sICAM-1 and sVCAM-1. Conversely, no significant associations were found between plasma IL concentrations and telomere length. However, the limited concentration range of some of the IL in this study does not preclude their biological effects on telomere dynamics. Taken together, the findings from this study suggest that increased systemic inflammation, consistent with vascular injury, is associated with decreased leukocyte telomere length. Future prospective studies would benefit from larger sample sizes with a balanced study design in order to discern subtle effects. Additionally, extended follow-up periods with equally spaced repeated measures would be beneficial in accurately assessing effect modification by inflammatory status on the rate of telomere attrition.
